# Suicide attempts and behavioral correlates among a nationally representative sample of school-attending adolescents in the Republic of Malawi

**DOI:** 10.1186/s12889-016-3509-8

**Published:** 2016-08-19

**Authors:** Masood A. Shaikh, Jennifer Lloyd, Emmanuel Acquah, Karen L. Celedonia, Michael L. Wilson

**Affiliations:** Centre for Injury Prevention and Community Safety (CIPCS), PeerCorps Trust Fund, 352/64 Makunganya Street, Co-Architecture Building, 4th Floor, P.O. Box 22499, Dar es Salaam, Tanzania

**Keywords:** Suicide attempts, Adolescents, Self-harm, Epidemiology, Africa

## Abstract

**Background:**

Suicide is among the top causes of adolescent mortality worldwide. While correlates of suicidal behavior are better understood and delineated in upper-income countries, epidemiologic knowledge of suicidal behavior in low-income countries remains scant, particularly in the African continent. The present study sought to add to the epidemiologic literature on suicidal behavior in Africa by examining the behavioral correlates of suicide attempts among Malawi adolescents.

**Methods:**

A cross-sectional study using a nationally-representative sample extracted from publically-available data was conducted. Bivariate and multivariate analyses were performed to discern associations between suicide attempts and a host of behavioral variables. 2225 records were included in the study.

**Results:**

At the multivariate level, suicide attempters had significantly higher odds of being anxious, being physically bullied, having sustained a serious injury and having a greater number of lifetime sexual partners. Alcohol use (at an early age and within the past 30 days) was also associated with suicide attempts.

**Conclusions:**

These findings have the potential to guide public health interventions geared toward suicide prevention in Africa and other, similar regions, as well as provide the impetus for future epidemiologic studies on suicidal behavior in low-income countries.

## Background

Global patterns in adolescent mortality have shifted during the past two decades from predominately infectious to social etiologies. Injuries (both intentional and unintentional) are among the leading causes of death among young people in nearly every region of the world [1]. Completed suicide is considered an intentional injury and recent research documents that suicidal behaviors may be on the rise [2, 3].

Suicide is a multifaceted issue and deaths which happen as a result of suicide are only part of the problem. Suicide ideation, which can range from thoughts of suicide to the actual planning of a suicide attempt, has a global prevalence of 15–25 % among adolescents [4]. Thoughts of suicide are often accompanied by symptoms of harmful internalizing behaviors such as depression and anxiety [5]. A significant proportion of nonfatal suicide attempts result in traumatic brain injuries or other potentially permanently disabling or disfiguring outcomes [6, 7]. Suicide attempts among adolescents vary by sex, with females attempting suicide at higher rates on average (1.3–3.8 % in males and 1.5–10.1 % in females) [4]. The risk of a person attempting a suicide is higher in the year after the first suicide attempt (5–15 %) and the most important risk factor for suicide is a prior suicide attempt [4, 8].

Studies among adolescents in low- and upper-middle income countries present a general trend of higher suicide ideation in low-income countries (LIC) compared to upper middle-income countries (UMIC): 27.9 % in Kenya, and 21.9 % in Benin (both LIC) compared to 17.5 % in Seychelles and 10.2 % in Costa Rica (both UMIC) [9, 10]. Poverty and reduced opportunities for self-determination are among the myriad of factors which have shown to be associated with adolescent suicidal behaviors [11]. These factors are particularly pronounced in LICs. Even in more affluent settings such as the USA, an individual’s socio-economic standing relative to others strongly influences their mental health [12, 13].

In much of the global south, suicide is a silent epidemic [14]. The problems that can precede suicide are not often visible, the actual number of suicide attempts is not known, and the public is largely unaware of the impact of suicide or the importance of mental health services in preventing suicide [15, 16].

In all settings, suicidal behavior has a number of underlying risk factors which are complex and interact with one another [17]. In the African context, psychosocial factors, specifically those related to psychosocial distress, are among the driving factors in suicide ideation [18]; which could be due to the difficult life circumstances that adolescents in African settings experience [14]. These include the ongoing HIV/AIDS epidemic leaving many youth of adolescent age orphaned and homeless, high unemployment rates and crippling poverty [18].

The aim of the present study was to examine self-reported suicide attempts and behavioral correlates of suicidal behavior among a nationally-representative sample of school-attending adolescents in the Republic of Malawi, a southern African country.

## Methods

### Setting

The data for this study were collected in the Republic of Malawi. Malawi is a land-locked south-eastern African country with a Human Development Index rank of 170 out of 186 according to 2011 estimates. It has a population of 15.38 million and a life expectancy of approximately 54 years with almost half of the population aged 0–14 years [19, 20]. In male and female adults aged 15 years and older, the literacy rate was 61.3 % in 2010 [21]. The under-five mortality rate in 2010 was 90.9 per 1,000 people and is steadily decreasing and the maternal mortality ratio has leveled off around 629.0 deaths per 100,000 live births [21]. The life expectancy at birth was 53.5 years for females and 53.4 years for males [21]. On average, 67.7 % of males and females combined completed primary school education; 69.0 % for females and 66.4 % for males [21]. Education is compulsory for primary school, children ages 6 to 14, and, on average, only 29.7 % of males and 28.8 % of females enroll in secondary school (http://www.classbase.com/Countries/Malawi/Education-System, http://www.unicef.org/infobycountry/malawi_statistics.html). In Malawi, 38.8 % of the population has access to improved sanitation facilities compared to 28.2 % in other Sub-Saharan African countries [21]. In Malawi, 11.7 % of people aged 15–49 years old were HIV positive compared to an average of 4.8 % in other Sub-Saharan African countries [21].

### Sample

We used publicly available data from Malawi obtained through the 2009 Global School-based Student Health Survey (GSHS) and conducted a secondary analysis with the major aim of analyzing behavioral covariates for suicide attempts. The World Health Organization (WHO) in collaboration with United States Centers for Disease Control (CDC) developed the methodology for the GSHS. This survey is administered to school attending adolescents and collects self-reported information on indices pertaining to health risk behaviors and practices. Countries were able to develop a questionnaire unique to their country. In Malawi, 50 government primary schools participated in the survey and 2,359 students in grades (‘Standards’) 7 and 8 aged 11 – 16 years (53.4 % females) participated in the survey with a 100 % school response rate, and a 94 % student response rate [22]. The GSHS employed a two-stage cluster sample design in order to produce a nationally representative dataset. In our analyses presented in this paper, we excluded 134 records with missing age and/or sex information.

No student or school identifiers are included in any of the public use data sets. The Ministry of Health in Malawi conducted the survey and at the time of data collection the study was approved at the national level by the Health Research Ethical committee of the Ministry of Health in Malawi. Consistent with the GSHS study protocol [22], questionnaires were administered to all eligible participants in an anonymous, voluntary manner. Written permission had been obtained from each participating school and from all classroom teachers. Parental consent was also obtained.

### Measurements

The dependent variable ‘suicide attempts’ was derived from one question in the GSHS: “*During the past 12 months, how many times did you actually attempt suicide?*”, with response options of “*0 times*”, “*1 time*”, “*2 or 3 times*”, “*4 or 5 times*”, and “*6 or more times*”. These responses were dichotomized into ‘zero’ corresponding to “*0 time*” and the rest were grouped together as ‘1’, representing those students who attempted one or more acts of suicide during the 1 year preceding the survey.

For the independent variables we selected contextually pertinent demographic, family characteristics and personal behaviors that have been either associated with suicide attempt in previous studies or constituted involvement in risk taking behaviors linked with poorer mental health. These included age in years, sex, bullying of any form, involvement in physical fights, food deprivation, having been physically attacked, age at sexual debut in years, serious injuries, physical bullying, loneliness, anxiety, suicidal ideation, suicide planning, number of close friends, tobacco use by parents, smoking by other people in the presence of the respondent, and number of lifetime sexual partners. Details on how these variables were created can be found in Table 1.

**Table 1:** Independent variable derivation from GSHS survey data 2009

Survey question	Coding	Variable
Demographic variables		
How old are you?	11 – 16 years (coded categorical	Age
What is your sex?	Male (1)	Sex
	Female (0)	
Dependent variable		
During the past 12 months, did you ever seriously consider attempting suicide?	No (0)	Suicide ideation
	Yes (1)	
During the past 12 months, did you make a plan about how you attempt suicide?	No (0)	Suicide planning
	Yes (1)	
Independent variables		
During the past 12 months, how often have you been so worried about something that you could not sleep at night?	Most of the time/always (yes)	Anxiety
	Never/rarely/sometimes (no)	
During the past 12 months, how often have you felt lonely	Most of the time/always (yes)	Loneliness
	Never/rarely/sometimes (no)	
During the past 30 days, how often did you go hungry because there was not enough food in your home?	Most of the time/always (yes)	Food deprivation
	Never/rarely/sometimes (no)	
How many close friends do you have?	0, 1, 2, and 3 or more friends (coded continuous)	Close friends
During the past 30 days, on how many days were you bullied?	3 or more days (bullied)	Bullying victimization
During the past 30 days, how were you bullied most often?	I was hit, kicked, pushed, shoved around, or locked indoors (physical bullying)	Physical bullying
Which of your parents or guardians use any form of tobacco?	Father, mother, or male/female guardians (yes)	Parental tobacco use
	Any other (no)	
During the past 7 days, on how many days have people smoked in your presence?	0 days (0)	Days people smoked in presence during the last week
	1–2 days (1)	
	3–4 days I(32)	
	5–7 days (4) (coded continuous)	
During the past 12 months, how many times were you in a physical fight?	0 – 1 times (no)	Physical fight
	2 – 12 or more times (yes)	
During the past 12 months, how many times were you seriously injured?	0 – 1 times (no)	Serious injury
	2 – 12 or more times (yes)	
How old were you when you had sexual intercourse for the first time?	Never having sexual intercourse and having sexual intercourse at age 15 or higher (no)	Early sexual debut
	Other responses (yes)	
During your life, with how many people have you had sexual intercourse?	0 – 6 people (coded continuous)	Lifetime sexual partners
How old were you when you had your first drink of alcohol other than a few sips?	Never had an alcoholic drink and having it at age 16 and older (no use of alcohol)	Alcohol use at early age
	Other responses (use of alcohol)	
During the past 30 days, on how many days did you have at least one drink containing alcohol?	0 days (no) 1 – 30 days (yes)	Alcohol use in the past 30 days

### Statistical Analysis

Analyses entailed univariate and bivariate analyses. Based on the results of the bivariate analyses, multivariate analyses were performed. Initially, distribution of each selected variable was examined within both categories of suicide, i.e. whether suicide was attempted or not. Crude and adjusted Odds Ratios (OR) along with their 95 % confidence intervals are reported for the strength and direction of associations between suicide and the factors studied. Stata version 12 (StataCorp, College Station, TX, USA, 2011) and the R Statistical Environment (R Development Core Team, 2011) [23] were used for the data analyses.

## Results

The mean age of the total sample was 13.9 years (SD = 0.9). There were no significant differences in the mental health or violence categories between rural and urban students. During the recall period of 12 months, 12.9 % of school attending adolescents reported having a suicide attempt at least once: 7.15 % of adolescents reported having one suicide attempt, while 5.75 % percent reported having made two or more such attempts. There was a preponderance of females among suicide attempters, 55.0 % versus 45.0 % among males, however this difference was not statistically significant (*p*-value: 0.51). Suicidal ideation was reported by 12.8 % of respondents (Males: 39.2 %, Females: 60.8 %; *p*-value: < 0.01) while suicidal planning was reported by 20.7 % (Males: 39.6 %, Females: 60.4 %; < 0.01) in the past 12 months.

Having been bullied in the past 30 days was reported by 33.9 % among suicide attempters, while having been physically bullied was reported by 13.4 % in this group (Table 2). In the past 12 months, involvement in physical fights was reported by 20.7 % of suicide attempters; 38.4 % reported having been physically attacked; while 52.5 % reported having sustained a serious injury during a 12 month period. Suicide attempters were also more likely to report being lonely and anxious in the past 12 months, 27.5 and 27.5 %, respectively, than non-suicide attempters.

**Table 2:** Comparison of factors by suicide attempt among 2225 school-attending adolescents in Malawi, GSHS 2009

Variable	No suicide attempt (*n* = 1,938)	One or more suicide attempts (*n* = 287)	*P*-value
Age (SD)	13.9 (0.89)	14.1 (0.87)	<0.001
Lifetime sexual partners (SD)	1.3 (0.89)	1.6 (1.2)	<0.001
Bullied	16.5	33.9	<0.001
Physical fight	8.1	20.7	<0.001
Sex (male)	47.0	45.0	0.514
Food deprivation	11.5	17.6	0.003
Physically attacked	17.0	38.4	<0.001
Early sexual debit	10.9	23.9	<0.001
Serious injury	27.4	52.5	<0.001
Physically bullied	5.4	13.4	<0.001
Loneliness	14.6	27.5	<0.001
Anxiety	10.8	27.1	<0.001
Suicide ideation	9.1	38.7	<0.001
Suicide planning	16.3	53.9	<0.001
Parental tobacco use	11.0	27.7	<0.001
Close friends			0.059
0	8.2	13.0	
1	34.2	32.6	
2	27.3	27.2	
≥ 3	30.3	27.2	
Use of alcohol at early age	7.3	21.3	<0.001
Use of alcohol in past 30 days	4.0	16.2	<0.001

All variables are expressed as proportions (in %) with the exception of age and life time sexual partners (mean and standard deviation)

Table 2 shows the crude distribution of all selected factors disaggregated by presence or absence of suicide attempt.

Factors found to be statistically significantly associated with suicide attempt in the bivariate analysis were used for multivariate logistic regression analysis. Table 3 shows the association between suicide attempt and the adjusted odds ratios (AOR) of covariates, along with 95 % confidence intervals (CI). Suicidal planning was more likely to be reported by suicide attempters (AOR = 4.50; 95 % CI = 1.82– 11.12) than non-suicide attempters. They were also more likely to have sustained serious injury (AOR = 3.62; 95 % CI = 2.47– 5.31), experienced being physically bullied (AOR = 2.78; 95 % CI = 1.41– 5.49), have anxiety (AOR = 3.55; 95 % CI = 1.80– 7.0), and had more lifetime sexual partners (AOR = 1. 59; 95 % CI =1.02– 2.48) than non-suicide attempters.

**Table 3:** Outcomes of multivariate analysis of variables associated with suicide attempts among school-attending adolescents in Malawi, GSHS 2009

Variable	Adjusted Odds Ratio	95 % Confidence Interval	*P*-value
Age	1.38	0.96, 1.98	0.085
Lifetime sexual partners	1.59	1.02, 2.48	0.042
Bullied	1.11	0.56, 2.21	0.771
Physical fight	0.64	0.19, 2.15	0.471
Food deprivation	0.55	0.21, 1.43	0.220
Physically attacked	1.37	0.56, 3.37	0.487
Early sexual debut	0.26	0.04, 1.89	0.184
Serious injury	3.62	2.47, 5.31	<0.001
Physically bullied	2.78	1.41, 5.49	0.003
Loneliness	1.56	0.83, 2.92	0.169
Anxiety	3.55	1.80, 7.00	<0.001
Suicide ideation	2.28	0.79, 6.58	0.126
Suicide planning	4.50	1.82, 11.12	0.001
Parental tobacco use	1.44	0.66, 3.15	0.354
Days people smoked in presence during past week	1.13	0.83, 1.53	0.436
Alcohol use at early age	0.83	0.23, 2.96	0.774
Alcohol use in past 30 days	8.04	0.97, 66.38	0.053

Only those factors found statistically significant in bivariate analysis were used in this model

Table 4 shows multivariate models adjusted for age and sex. With the exception of food deprivation and having close friends, all factors were statistically significantly positively associated with suicide attempt in the past 12 months. Highest adjusted odds ratios were found for early age at first alcohol use (AOR = 10.01; 95 % CI = 3.71– 27.01), alcohol use in the past 30 days (AOR = 13.70; 95 % CI = 5.98– 31.37), suicide planning (AOR = 5.95; 95 % CI = 2.91– 12.22), suicide ideation (AOR = 7.69, 95 % CI = 4.76– 12.44),anxiety (AOR = 4.47, 95 % CI = 3.19– 6.27) and early sexual debut (AOR = 3.73, 95 % CI = 1.96– 7.11). Having any close friends had a statistically significant protective effect against suicide attempts.

**Table 4:** Multivariate analysis of variables associated with suicide attempts among school-attending adolescents in Malawi, adjusted for age and sex

Variable	Adjusted Odds Ratio	95 % Confidence Interval	*P*-value
Lifetime sexual partners	1.66	1.28, 2.16	<0.0001
Bullied	2.76	1.62, 4.69	<0.001
Physical fight	2.97	1.66, 5.31	<0.001
Food deprivation	0.88	0.52, 1.48	0.631
Physically attacked	2.92	1.89, 4.53	<0.001
Early sexual debut	3.73	1.96, 7.11	<0.001
Serious injury	3.58	2.75, 4.65	<0.001
Physically bullied	3.34	1.63, 6.86	0.001
Loneliness	2.56	1.55, 4.24	<0.001
Anxiety	4.47	3.19, 6.27	<0.001
Suicide ideation	7.69	4.76, 12.44	<0.001
Suicide planning	5.95	2.91, 12.22	<0.001
Parental tobacco use	3.54	2.01, 6.25	<0.001
Days people smoked in presence during past week	1.24	1.07, 1.44	0.005
Close Friends			
0	1		
1	0.46	0.26, 0.80	0.006
2	0.45	0.28, 0.72	0.001
≥ 3	0.35	0.21, 0.59	<0.001
Alcohol use at early age	10.01	3.71, 27.01	<0.001
Alcohol use in past 30 days	13.70	5.98, 31.37	<0.001

Estimates adjusted for age and sex

## Discussion

We found that suicide attempt among school-based adolescents in Malawi (12.9 %) was lower than among other countries in the SSA region (17.5 %– 31.1 %) among youth of comparable age with similar recall periods [24, 25], but analogous to those in high income country settings (12.7–19.0 %) [9, 26]. Higher elements of happiness and overall life satisfaction in Malawi relative to other countries in Africa may contribute to these differences [27]. In 2006 Malawi took part in the Mchinji Social Cash Transfer Pilot in order to provide improved access to basic necessities and there have been recognized advances in school enrollment, better preparedness against natural and economic disasters, and improved access to healthcare [27]. All of the above may contribute to the lower rate of self-reported suicide attempts among school-attending adolescents in Malawi.

All analyzed covariates were found to be significantly positively associated with suicide attempts except for food deprivation and having close friends. Early age at first alcohol use, alcohol use within the past 30 days, suicide ideation, suicide planning, anxiety and early sexual debut had the highest odds of suicide attempt among school-attending adolescents in Malawi which were consistent with findings from other studies [28, 29]. This study found that having any close friends, whether that is one or many, had a protective effect against suicide attempts. It is well known that social isolation is associated with poor psychological health including suicide attempts and low self-esteem [30, 31]. These results are consistent with results from GSHS surveys in other Sub Saharan African countries showing that better programs addressing psychological health would decrease the prevalence of suicide attempts, as well as substance abuse, among adolescents [24, 32].

Interestingly, females are more likely to have suicidal ideation, planned and attempted suicide than males. These findings are not consistent with others elsewhere in the region [18, 24, 33, 34], but they are consistent with international studies [35]. It is known that gender specific research finds females more likely to think about and attempt suicide, while males have higher suicide rates [33, 35]. Early age of sexual debut may play a large role in this as it was significantly correlated with suicide attempts in this study. HIV/AIDS may play a large role in this due to the fact that 58.0 % of the infections in Malawi are among young girls in the 15–24 year age range [36]. While the country is trying to improve the collective socio-economic well-being of its youth via the Cash Transfer Pilot, rates of poverty remain considerable and girls as young as 15 resort to selling sex as supplementary income which puts them at even greater risk of trafficking, sexual exploitation, sexually transmitted infections, and suicidal behaviors [36].

This study found that 13.4 % of the sampled adolescents who had attempted one or more acts of attempted suicide reported being physically bullied; a rate considerably lower than those found among other studies in Africa [25, 37]. However, consistent with other studies, being physically bullied was positively associated with suicide attempt and suicidal ideation [25, 38]. Being the victim of physical bullying can precipitate other psycho-social problems such as alcohol use, anxiety and loneliness, all of which are correlated with increased risk for attempted suicide in this study [39].

Food deprivation was not found to be statistically significantly associated with suicide attempts. There are many possible explanations for this; for one, the percent of children experiencing food deprivation were much lower than most of the other variables analyzed leading us to the conclusion that food deprivation is not a significant problem in the study population. Food insecurity is linked to poverty and the lack of resources to break out of it, thus it is possible that children who experienced severe food deprivation had to work to help their parents and could not afford to go to school and so were not included in the study [40].

The results of this study must be viewed in light of its limitations. First, this study was conducted through secondary analysis and so there are limitations to the data that is collected. Studies such as this do not prove causation, only provide information on correlations. As with a similar study conducted in the Seychelles, this study did not include suicide attempts among school-going adolescents who were absent on the day of the survey which contributes to selection and information bias [33]. Previous surveys have documented that adolescents who are absent from school displayed higher risks of suicidal behavior than those who attended on a regular basis [41]. Another limitation is the cultural stigmatization of mental health problems in most of the region, including suicide, which makes individuals less likely to seek help or admit to past attempts potentially leading to an underreporting bias. Recall bias may have played a role in underreporting or over reporting as well since the recall period in the questionnaire was 12-months.

This study provides useful insight into an important public health issue that has rarely been studied among adolescents in African settings. To our knowledge, this is the first study to report on suicide attempts using a nationally representative sample of school-attending adolescents in the region. Several factors have helped to ensure the validity of the present findings. The large sample size combined with minimal missing data has enabled robust analyses and conclusions which are generalizable to the country of Malawi. The questionnaires were anonymous which minimizes bias in the form of responses which are socially desirable. The questionnaire used in this study has been used in several other settings in the region [18, 41, 42].

## Conclusions

Our results suggest that while suicidal attempts among school-going adolescents in Malawi are lower than among other countries in the region, adolescents in the country would benefit from more school programs targeting specific factors which underlie suicidal behaviors, particularly substance abuse and psychological issues. At the time of this study, the authors could not find any examples of a national or community-based strategy aimed at suicide prevention in Malawi. Health professionals should focus on addressing suicidal ideation, bullying, and alcohol use when designing suicide prevention programs specific to this age group. Health promotion programs should also provide information tailored specifically to young girls on developing positive attitudes to help diminish their inclinations toward suicidal ideation and attempts [18]. Greater outreach is needed to properly inform adolescents and their families in Malawi about the extent of the problem of suicidal behavior and to identify appropriate next steps to recognize and prevent suicidal behavior. The findings here may also be useful in enhancing programs which highlight the importance of de-stigmatizing mental illness in all its forms in the African region.
